# Secondary Tumors of the Pancreas: A Multicenter Analysis of Clinicopathological and Endosonographic Features

**DOI:** 10.3390/jcm12082829

**Published:** 2023-04-12

**Authors:** Marco Spadaccini, Maria Cristina Conti Bellocchi, Benedetto Mangiavillano, Alberto Fantin, Daoud Rahal, Erminia Manfrin, Francesca Gavazzi, Silvia Bozzarelli, Stefano Francesco Crinò, Maria Terrin, Milena Di Leo, Cristiana Bonifacio, Antonio Facciorusso, Stefano Realdon, Chiara Cristofori, Francesco Auriemma, Alessandro Fugazza, Luca Frulloni, Cesare Hassan, Alessandro Repici, Silvia Carrara

**Affiliations:** 1 Endoscopic Unit, Department of Gastroenterology, IRCCS Humanitas Research Hospital, Via Manzoni 56, 20089 Milan, Italy; marcospadaccini9@gmail.com (M.S.);; 2 Department of Biomedical Sciences, Humanitas University, Via Manzoni 113, 20089 Milan, Italy; 3 Gastroenterology and Digestive Endoscopy Unit, The Pancreas Institute, G.B. Rossi University Hospital, 37134 Verona, Italy; 4 Digestive Endoscopy Unit, Division of Gastroenterology, Humanitas Mater Domini, 21053 Castellanza, Italy; 5 Gastroenterology Unit, Istituto Oncologico Veneto IOV-IRCCS, 35128 Padova, Italy; 6 Department of Pathology, IRCCS Humanitas Research Hospital, Via Manzoni 56, 20089 Milan, Italy; 7 Pancreatic Surgery Unit, IRCCS Humanitas Research Hospital, Via Manzoni 56, 20089 Milan, Italy; 8 Medical Oncology and Hematology Unit, Humanitas Cancer Center, IRCCS Humanitas Research Hospital, Via Manzoni 56, 20089 Milan, Italy; 9 Digestive Endoscopy Unit, Division of Gastroenterology, San Paolo Hospital, 20090 Milan, Italy; 10 Department of Radiology, IRCCS Humanitas Research Hospital, Via Manzoni 56, 20089 Milan, Italy; 11 Gastroenterology Unit, Department of Surgical and Medical Sciences, University of Foggia, 71122 Foggia, Italy

**Keywords:** pancreas, cancer, oncology, surgery, metastasis

## Abstract

Many tumors may secondarily involve the pancreas; however, only retrospective autopic and surgical series are available. We retrospectively collected data from all consecutive patients with histologically confirmed secondary tumors of the pancreas referred to five Italian centers between 2010 and 2021. We described clinical and pathological features, therapeutic approach and treatment outcomes. EUS characteristics of the lesions and the tissue acquisition procedures (needle, passages, histology) were recorded. A total of 116 patients (males/females 69/47; mean age 66.7) with 236 histologically confirmed pancreatic metastases were included; kidney was the most common primary site. EUS was performed to confirm the diagnosis in 205 lesions which presented as predominantly solitary (59), hypoechoic (95) and hypervascular (60), with a heterogeneous (*n* = 54) pattern and well-defined borders (*n* = 52). EUS-guided tissue acquisition was performed in 94 patients with an overall accuracy of 97.9%. Histological evaluation was possible in 88.3% of patients, obtaining final diagnosis in all cases. When cytology alone was performed, the final diagnosis was obtained in 83.3% of cases. A total of 67 patients underwent chemo/radiation therapy, and surgery was attempted in 45 (38.8%) patients. Pancreatic metastases are a possible event in the natural history of solid tumors, even long after the diagnosis of the primary site. EUS-guided fine needle biopsy may be suggested to implement the differential diagnosis.

## 1. Introduction

Many extrapancreatic tumors may secondarily involve the pancreas, with an incidence ranging from 3% to 12% [1,2] and a broad spectrum of both clinicopathological features and outcomes. Knowledge is still lacking about the molecular pathways and the anatomical reasons supporting the behavior of some of the most common tumors associated with pancreatic metastasis, such as renal cell carcinoma and melanoma [3,4].

Most evidence is based on retrospective analysis of autopsies and surgical series, due to the high incidence (around one-third of secondary pancreatic tumors) of lesions clinically mistaken as primary pancreatic tumors before surgical resection [1]. For these reasons, those series could not provide data on how to improve accuracy during the diagnostic work-up. 

Considering the morbidity (and mortality) related to pancreatic surgery, pancreatic resections should be performed only when they are clinically indicated. As a matter of fact, among other challenges, in the differential diagnosis of a pancreatic mass, the possibility of a metastatic lesion should always be considered. In this regard, accurate oncological anamnesis, extensive background knowledge of malignancies possibly involving the pancreas through metastasis and a precise imaging-based diagnosis of a pancreatic mass, particularly in a patient with concomitant or previous history of extra-pancreatic cancer, are mandatory. However, even if fundamental in raising the suspicion, clinical, epidemiological and radiological data are often useless in distinguishing between primary pancreatic cancer and metastases. 

As a consequence, the role of endoscopic ultrasound (EUS) in the diagnostic work-up of pancreatic masses has already been demonstrated and the EUS-guided tissue acquisition is of paramount importance for obtaining a reliable diagnosis [5], even if no data are available about the role of core needles for histology (over cytology) assessment.

The aim of this study was to describe clinical, endosonographic and pathological features of secondary tumors of the pancreas, along with their therapeutic approach and related outcomes.

## 2. Materials and Methods

### 2.1. Patients

A retrospective review of prospectively maintained databases of EUS procedures was carried out to identify all patients referred to the Endoscopic Units of five Italian centers (Humanitas Bergamo, Castellanza, and Rozzano; Policlinico GB Rossi Verona; Istituto Oncologico Veneto, Castelfranco Veneto) between April 2010 and April 2021. Data from patients with a histologically confirmed diagnosis of secondary pancreatic tumors were retrieved for the study analysis. Furthermore, we performed a chart revision of all patients who underwent pancreatic resections for secondary pancreatic malignancies in the five centers during the same timeframe.

Patients with locally advanced non-pancreatic primary tumors that involved the pancreas by direct extension (e.g., pancreatic infiltration by left kidney cancer) were excluded.

Demographic and clinical characteristics were collected. In particular, the time of recurrence was defined as the time (in months) between the first radiographic remission of the primary neoplasia and the histological diagnosis of metastasis into the pancreatic parenchyma. Treatment and outcome data were collected as well.

The study was conducted according to the Declaration of Helsinki and approved by the institutional review board (IRB) of the coordinating center (Humanitas Research Center, Rozzano). All the patients gave their consent to the procedures and to the research purposes. The study protocol was registered at Clinicaltrial.gov (NCT02855151).

### 2.2. Endoscopic Ultrasound Procedures

The Olympus GF-UCT180 series linear array echoendoscope (Olympus Europa SE & CO. KG, Hamburg, Germany) in combination with the new EU-ME2 echoprocessor (Olympus SE & CO. KG, Hamburg, Germany) or Pentax EG-3870UTK linear echoendoscope (Pentax Medical, Hamburg, Germany) in combination with a Hitachi ultrasound machine were used.

Fine-flow (Pentax) or H-Flow (Olympus) were used to enhance the micro-vascularization of the masses: when a mass is kept very close to the tip, this modality shows high resolution details of the vascular pattern of the lesion. To define the vascular pattern of the lesion, contrast-enhanced EUS with intravenous contrast agent administration (Sonovue™, Bracco Imaging, Milan, Italy) was performed, as per centers’ shared protocol since 2015.

For each patient, EUS characteristics of the lesions were collected: size, location, number of focal masses, echotexture and vascularization.

Adverse events were recorded. Eloubeidi et al. [6] defined an adverse event as any deviation from the expected clinical course during or after EUS, related to the procedure.

EUS-guided tissue acquisition was performed with 22-gauge, 25-gauge or 19-gauge needles (Expect™ Slimline, Acquire™, Boston Scientific, Boston, MA, USA; Beacon bnx^®^, SharkCore Needle™, Medtronic, Newton, MA, USA), chosen at the discretion of the endosonographers. The biopsies were performed combining the fanning technique and the slow-pull technique [7,8]. In general, especially for hypervascularized lesions, no suction was applied, in order to reduce the contamination of the specimen with blood.

At each pass, if a micro-fragment or “worm-like” material was observed, it was placed in a container of 10% neutral buffered formalin fixative for the final histological examination and immunohistochemistry (IHC) staining. If drop-like material was obtained, it was smeared between 2 glass slides, fixed with ethanol and stained with a Papanicolaou stain for cytological analysis.

The therapeutic path was always decided through multidisciplinary team (MDT) discussion involving endoscopists, surgeons, oncologists, radiation oncologists and radiologists. The surgical methodology is reported in the Appendix A.

### 2.3. Pathological Evaluation of EUS and Surgical Specimens

After 24 h fixation in 10% buffered formalin, all the biopsy specimens were stained with haematoxylin and eosin (H&E) and the slides were evaluated by expert pathologists specialized in Hepato-Bilio-Pancreatic disease. Ancillary analysis such as immunohistochemistry (IHC) staining was performed if the pathologist deemed it was necessary to better define the histological diagnosis and grading and in the case of hormone-secreting tumors.

The surgical specimens were observed by the pathologist and a gross description was made: size, site and number of lesions were recorded in the pathological report.

### 2.4. Statistical Analysis

Data were entered into an Excel spreadsheet (Microsoft Excel 2010; Microsoft Corporation, Redmond, WA, USA). All continuous variables were described as mean and standard deviation, while dichotomous variables were reported as percentage. Statistical analysis was performed using SPSS version 17 (SPSS Inc., Chicago, IL, USA).

## 3. Results

### 3.1. Baseline Characteristics

**Figure 1:**
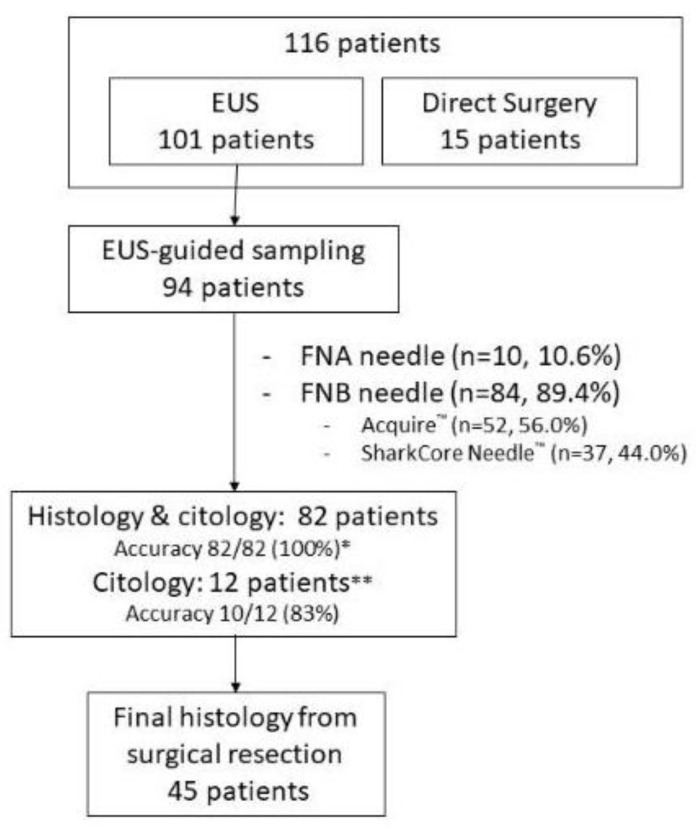
Study flowchart. * histology only accuracy 81/82 (98.8%), cytology only accuracy 73/82 (89.0%); ** no core obtained, but material adequate for cytological diagnosis.

A total of 116 patients (male/female: 69/47; mean age: 66.7 ± 10.1 years—range: 26–86 years) with 236 histologically confirmed pancreatic metastases were included in the analysis (Figure 1). 

Lesion distribution was relatively even among pancreatic head, uncinated process, neck, body and tail, and the mean lesion size was 25.4 ± 15.2 mm, ranging from 2.7 to 90.0 mm (Table 1).

The most common primary neoplastic site was the kidney (75 cases), with all of the histology types being clear cell renal carcinoma. In 40 out of 75 cases (53.3%), the pancreas was the unique metastatic site. The other most common primary sites were the colon (*n* = 9), breast (*n* = 7), lung (*n* = 7) and melanoma (*n* = 7). In addition, there were three cases of fibro-leiomyosarcoma, three of ovarian cancer, two of liver cancer and one case of Merkel cell tumor, thyroid cancer and non-Hodgkin lymphoma. In most of the non-kidney cancers (37/41, 90.2%), the pancreas was not the only metastatic site.

In seven cases, the lesions were found during the initial staging work-up. Among the other 109 patients, pancreatic metastases were diagnosed after a mean time of 97.8 ± 79.4 months, with nearly two-thirds of cases (*n* = 70, 60.3%) having a latency interval of at least 5 years. Of note, no cases of lung metastasis were found after more than two years from the initial diagnosis. In most of the cases (*n* = 94), the lesions were asymptomatic, which were incidental findings during the staging/follow-up. Nine lesions caused jaundice and pancreatic-like pain was reported in seven cases. Further, weight loss, anemia and asthenia were reported by a minority of patients (*n* = 6).

### 3.2. EUS Characteristics

**Figure 2:**
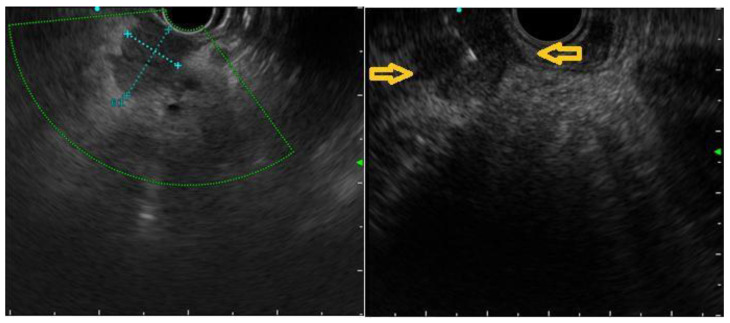
Breast cancer metastasis to pancreas. Yellow arrows point at margins’ irregularity.

In 101 patients, an EUS was performed in order to confirm the diagnosis of 205 pancreatic lesions with a mean size of 23.8 ± 15.3 mm, ranging from 2.7 to 73.0 mm. In most of the cases, they were solitary (*n* = 59), hypoechoic (*n* = 95) lesions, with a heterogeneous (*n* = 54) pattern and well-defined borders (*n* = 52) (Figure 2). 

The vascular evaluation showed 60 hypervascular lesions, with a heterogeneous vascular pattern in 32 cases. EUS features per histology type are reported in Table 2 for main histology types.

EUS-guided fine needle sampling was successfully performed in 94 patients with an overall diagnostic yield of 97.9% (92/94) after an average number of 2.4 ± 1.3 needle passes (range: 1–4). In 82 out of 94 cases (88.3%), the presence of a “worm-like” core also allowed a histologic evaluation after formalin fixation, and the final diagnosis was obtained in all cases. In 9 of the 82 patients (11.0%) with availability of both cytological and histological specimens, the cytology alone had failed in reaching the diagnosis. Conversely, among the 12 cases in which no tissue core was retrieved because of the drop-like or bloody aspect of the material obtained, the final diagnosis was enabled by cytology assessment in 10 out of 12 cases (83.3%).

Immunostaining studies were performed on 78 out of 82 formalin-fixed histology samples (95.1%) and on 9 out of 33 cytology samples (27.3%).

### 3.3. Treatment and Outcomes

Sixty-five patients underwent chemotherapy and two patients underwent radiation therapy. In 45 patients (38.8%), the surgical approach was attempted (see surgical outcomes in Appendix A).

A total of 90 patients were followed up for a mean time of 25.3 ± 21.1 months and 16 of them died during the follow-up period (4 deaths due to unrelated causes). Among those still alive, 43 out of 74 patients were free from disease after a mean follow-up time of 24.9 ± 8.4 months. Eighteen patients were lost at follow-up.

## 4. Discussion

Most pancreatic lesions are primary neoplasms, and of these more than 90% are pancreatic ductal adenocarcinomas, but the pancreatic parenchyma may also be a site of metastases from other primary sites. As a matter of fact, secondary tumors of the pancreas can have an incidence rate ranging from 3% to 12% [2]. In particular, some tumors seem to favor the pancreas, and the biological mechanisms that sustain this interaction between the circulating tumor cells and the host organ have yet to be fully explained [9]. Thus, in a patient with a previous history of cancer and a new diagnosis of a pancreatic mass, determining whether the pancreatic lesion is a primary or secondary tumor is necessary in order to plan the best treatment. This is even more relevant considering the possible long interval time between primary tumor and metastasis diagnoses, ruling out any possibility of identifying any sort of “safe zone”. Indeed, in our series, secondary pancreatic tumors occurred after a mean period of more than 8 years, after the clinical and/or radiological remission of the primary tumor, with a maximum period of 17 years. Our findings corroborate the long time between the diagnosis and management of the primary tumor and the evidence of pancreatic metastasis, with a maximum latency period ranging from 14 months to 22 years [10] described in the literature. As a result, secondary tumors are often not the first hypothesis during an initial evaluation of the pancreatic masses. This is why, when a secondary neoplasm is suspected, a thoroughly detailed clinical history is of paramount importance in order to orient the diagnosis through adequate imaging and EUS-guided sampling, planning the right panel of immunohistochemical staining.

In the literature, the most common cancers with metastases to the pancreas include lung cancer, renal cell cancer, colon cancer, melanoma, sarcoma and breast cancer [11], but haematological malignancy metastases have also been described [12]. We described a prevalence of the kidney as the primary site of pancreatic secondary tumors, as described in previous studies [13,14,15,16,17,18]. However, an autopsy study from Japan reported gastric adenocarcinoma as the most common primary site [11], while in previous clinical series, more than a quarter of metastases to the pancreas had originated from the lung [1,19]. The different incidence in the primary site may be due to population-based differences, in particular for the Japanese study [11], but also to the patients analyzed (case series vs autopsy cases) and to a different prognosis of the primary neoplasm over time.

Symptoms of pancreatic secondary tumors are often absent and therefore they are identified during the initial work-up of the primary tumor or during routine surveillance after its resection. We reported 80% of asymptomatic patients as described in other series [14,20]. Unfortunately, when secondary tumors cause symptoms, they are similar to those reported for primary pancreatic cancer, such as abdominal pain and jaundice [21]. The similar clinical presentation and the long interval from primary neoplasm treatment make it difficult to differentiate the clinical diagnosis between primary pancreatic tumor and pancreatic metastasis.

Moreover, in half of our cases, the patients had only one pancreatic mass. In these cases, the distribution of metastases did not help to distinguish secondary tumors from primary pancreatic cancer, as stated in previous studies [5,13]. However, other series have reported the pancreatic head as the favored site of secondary tumors [22,23]. In this scenario, the role of EUS has become fundamental in order to reach a final diagnosis and choose the best treatment option for the patient. Noninvasive cross-sectional imaging (multidetector computed tomography and magnetic resonance imaging) can provide a general assessment of malignancy potential and resectability, and the presence of lymphadenopathy and/or of distant metastases [24]. However, the radiologic distinction between a primary and secondary pancreatic neoplasm is often limited, although enhanced computed tomography scans and magnetic resonance imaging may be contributory, especially if contrast medium is used [25,26,27].

**Figure 3:**
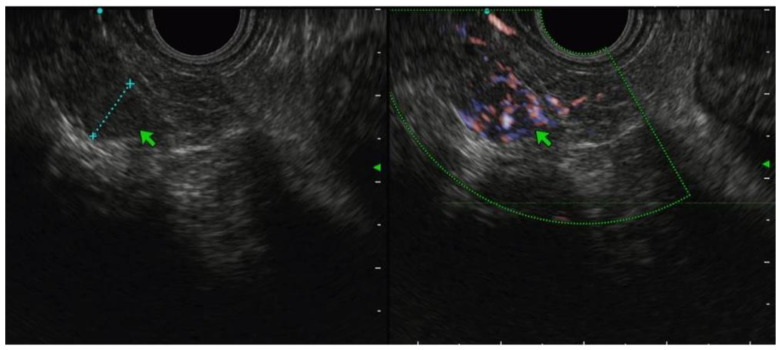
Renal cancer metastasis to pancreas. Green arrows point at margins’ irregularity.

The most prevalent EUS characteristics in our series were a heterogeneous hypoechoic pattern without cystic component and with well-defined borders. Our experience confirmed data from the literature, where the more common EUS characteristic of secondary pancreatic neoplasms are hypoechoic, hypervascular and masses with well-defined borders [14,20,28]. However, it also appeared clear that the endosonographic aspect of secondary pancreatic lesions alone fails to reliably distinguish among the different histologies and primary pancreatic cancer, with only kidney carcinoma having a stable EUS pattern (hypoechoic, hypervascular lesions with well-defined borders; Figure 3).

This underlines, on the one hand, the importance of always considering the possibility of looking at a pancreatic metastasis irrespective from the EUS specific patterns, and on the other hand, the option of EUS-guided sampling appears as an unmatched opportunity for pancreatic lesion diagnosis. As a matter of fact, even in our series, tissue acquisition is usually necessary to reach the final pathological diagnosis and to assess the site of origin of the mass. EUS biopsy is a safe, effective and efficient diagnostic tool in the evaluation of pancreatic masses. Cytopathological specimens, and more recently core biopsies, may be obtained with high sensitivity (75–98%), specificity (71–100%), positive predictive value (96–100%), negative predictive value (33–85%) and accuracy (79–98%) in the diagnosis of pancreatic cancer as compared to other modalities [29]. In agreement with the pathologist, a biopsy fragment should always be processed in a way that guarantees the better preservation of cells and tissues and reduces the risk to lose material, independently from the caliber and geometry of the needle used. In this regard, the main finding of our study is to highlight a potential benefit of using fine-needle biopsy (EUS-FNB) for its higher diagnostic yield and a higher possibility of immunohistochemical-based tests when compared to cytology, suggesting the choice of dedicated needles for adequate samples. FNB needles may therefore be preferred over FNA needles when available. Conversely, the relevance of both Rapid On-Site Evaluation (ROSE) and Macroscopic On-Site Evaluation (MOSE) in the case of suspected pancreatic metastasis needs to be assessed in further studies.

Our study is limited by a few drawbacks. First, the retrospective design prevents us from drawing unbiased conclusions. As a matter of fact, possible confounders in terms of lesion heterogeneity, clinical management and EUS protocol across the different centers cannot be ruled out. However, this large cohort represents one of the most comprehensive points of view on this underestimated issue. Secondly, we could report EUS data of only 205 among the 236 lesions (86.9%), since some of the lesions were directly referred to surgery after CT scan. In our opinion, such sharing of real-life data is aimed at increasing awareness of this issue. Interestingly, reports of pancreatic metastasis have been increasing in recent years [3], probably due to better accuracy of diagnostic examination and to the improvement in neoplastic disease outcomes with longer follow-up. In the near future, this may allow for the undertaking of a multicenter-based prospective effort in order to confirm the need for tissue sampling, and the superiority of EUS-FNB over fine-needle aspiration, in attempting to assess the optimal modality for an EUS-based approach. Moreover, another limitation of our study is that it failed in providing any hint about the reason why the pancreas is such a common site for specific tumor metastases, even if we may consider this beyond the purpose of this study. This topic has been diffusely investigated by previous studies; however, knowledge is still scarce, even for some of the most common tumors associated with pancreatic metastasis [3,4]. In particular, different mechanisms of occurrence of metastasis in renal cell cancer have been proposed; the exclusive pancreatic involvement we observed in several cases is difficult to reconcile with a systemic haematogenous seeding, especially when considering the small amount of blood flowing through the 120–180 g of pancreatic tissue. However, previous population-based studies attributed only little if any role to a local mechanism (i.e., lymphogenic or venous) [9], and further studies are still needed.

## 5. Conclusions

Metastases to the pancreas are a possible occurrence in the natural history of various solid tumors even long time after primary tumor diagnosis. EUS-guided tissue acquisition with fine-needle biopsy needles may be suggested to implement ancillary studies necessary for differential diagnosis.

## Figures and Tables

**Table 1:** Baseline characteristics. * 79 lesions >20 mm, 110 lesions 10–20 mm, 47 lesions < 10 mm.

**Patients (*n*)**	116
Male (*n*)	69
Mean age (years)	66.7 ± 10.1
**Primary tumor (*n*)**	
Kidney	75
Colon	9
Breast	7
Lung	7
Melanoma	7
Fibro-leiomyosarcoma	3
Ovarian cancer	3
Liver cancer	2
Merkel cell tumor	1
Thyroid cancer	1
Non-Hodgkin lymphoma	1
**Diagnostic timing (*n*)**	
Synchronous	7
Metachronous	109
**Symptoms (*n*)**	
Asymptomatic	94
Jaundice	9
Pancreatic-like pain	7
Weight loss	3
Anemia	2
Asthenia	1
**Lesions (*n*)**	236
Mean size (mm) *	25.4 ± 15.2
Location	
Head	57
Uncinated process	43
Neck	37
Body	50
Tail	49

**Table 2:** EUS features per histology type. Less than 3 patients with fibro-leiomyosarcoma, ovarian cancer, liver and thyroid cancer, Merkel cell tumor and non-Hodgkin lymphoma underwent EUS.

Histology (*n*)	Echogenicity	Echogenicity	Vascularization	Vascularization	Borders
		(Pattern)		(Pattern)	
Kidney (63)	Hypo = 59	Homo = 32	Hypo = 3	Homo = 46	Regular = 50
	Iso = 2	Etero = 31	Hyper = 60	Etero = 17	Irregular = 13
	Hyper = 2				
Colon (8)	Hypo = 8	Homo = 1	Hypo = 6	Homo = 6	Regular = 6
	Iso = 8	Etero = 7	Hyper = 2	Etero = 2	Irregular = 2
	Hyper = 8				
Lung (7)	Hypo = 6	Homo = 1	Hypo = 6	Homo= 3	Regular = 1
	Iso = 0	Etero = 6	Hyper = 6	Etero = 4	Irregular = 2
	Hyper = 1				
Breast (6)	Hypo = 6	Homo = 1	Hypo = 6	Homo = 3	Regular = 3
	Iso = 0	Etero = 5	Hyper = 0	Etero = 3	Irregular = 3
	Hyper = 0				
Melanoma (6)	Hypo = 6	Homo = 5	Hypo = 4	Homo = 3	Regular = 2
	Iso = 0	Etero = 1	Hyper = 2	Etero = 3	Irregular = 4
	Hyper = 0				

## Data Availability

The data are available upon request from the corresponding author.

## Appendix A. Surgical Procedures

Surgical interventions included total pancreatectomy, pancreaticoduodenectomy, distal pancreatectomy and enucleation. Demolition of the surgical specimen in total pancreatectomy was performed “en-bloc” to allow an accurate pathological examination. Reconstruction, when necessary, was realized with antecholic gastrojejunostomy. Pancreaticoduodenectomy was performed by end-to-side double-layer pancreaticojejunostomy, when duct-to-mucosa was possible. End-to-side hepaticojejunostomy was carried out 10–15 cm distal to the pancreatic anastomosis. A pylorus preservation procedure was usually performed, and an end-to-side ante-colic duodenojejunostomy was realized 30 cm distal to the biliary-enteric anastomosis. Distal pancreatectomy was usually performed according to radical antegrade modular pancreatosplenectomy (RAMPS) technique [9]. During surgical pancreatic enucleation, US was performed to confirm enough distance between lesion and Wirsung duct (≥3 mm).

Post-operative morbidity, mortality (death within 30 days after day of hospital discharge), reintervention and readmission rates were evaluated. Post-operative complications, including pancreatic, biliary, duodenal/gastric and lymphatic fistulas, abdominal abscess, delayed gastric emptying (DGE), wound infection and post-pancreatectomy haemorrhage (PPH) were graded according to the Clavien–Dindo Classification [10]. Post-operative pancreatic fistula (POPF), PPH and DGE were defined according to the International Study Group for Pancreatic Surgery recommendations [11,12,13].
